# AI-powered finite element analysis for predicting fracture patterns in endodontically treated teeth restored with posts

**DOI:** 10.6026/973206300213968

**Published:** 2025-10-31

**Authors:** Divya Batra, Priyatam Maruti Karade, Chirag R Vaniya, Ataul Hafeez Imran, Mohd Ahmed Ali Khan, Ishita Ghosh

**Affiliations:** 1 Department of Conservative dentistry and Endodontics, National Dental College and Hospital, Dera Bassi, Punjab, India; 2 Department of conservative dentistry and endodontics, Bharati Vidyapeeth (Deemed To Be University) Dental College and Hospital, Sangli, Maharashtra, India; 3 Department of Prosthodontics and Crown & Bridge, Government Dental College and Hospital, Jamnagar, Jamnagar, India; 4 Department of Conservative Dentistry and Endodontics, FICOI (USA), Fellowship Laser Dentistry (Wcli, USA) & Currently Practicing At Noor Hospital, Qadian, Gurdaspur, Punjab, India; 5 Conservative Dentistry and Endodontics, Rungta college of dental science and research, Hyderabad, India; 6 Department of Oral and Maxillofacial Surgery, Kalinga Institute of Dental Sciences, KIIT Deemed to be University, Bhubaneswar, Odisha, India

**Keywords:** Artificial intelligence, finite element analysis, endodontically treated teeth, fracture patterns, post systems, predictive modeling and dental biomechanics

## Abstract

Endodontically treated teeth (ETT) are prone to fracture due to structural compromise and conventional finite element analysis (FEA) has limitations in accurately predicting fracture behavior. Therefore, it is of interest to evaluate an artificial intelligence (AI)-enhanced FEA model for predicting fracture patterns in ETT restored with fiberglass, carbon fiber, zirconia and cast metal posts. Hence, a total of 120 maxillary premolars were tested, with the AI model trained on 500 prior FEA simulations and validated against experimental fracture resistance outcomes. The AI-powered FEA showed superior predictive accuracy (92.3%) compared to conventional FEA (76.8%) and closely correlated with actual fracture initiation sites (r = 0.91). Integration of AI with FEA enhances fracture prediction and may guide clinicians in selecting optimal post systems for improved outcomes in ETT.

## Background:

Endodontically treated teeth (ETT) present unique challenges in restorative dentistry due to their increased susceptibility to fracture following root canal treatment [1]. The removal of dentin during endodontic access and instrumentation, coupled with the loss of tooth structure from caries or previous restorations, significantly compromises the structural integrity of these teeth [2]. Consequently, ETT are approximately twice as likely to fracture compared to vital teeth, with fracture rates ranging from 1.6% to 19.9% depending on the restoration method and follow-up period [3]. The restoration of ETT with posts has been a cornerstone of endodontic restorative procedures for decades, providing retention for the final restoration and reinforcing the remaining tooth structure [4]. However, the selection of appropriate post materials and designs remains controversial, with various studies reporting conflicting outcomes regarding fracture resistance and failure patterns [5]. Traditional post systems include metal cast posts and cores, which have been largely replaced by fiber-reinforced composite posts due to their elastic modulus closer to dentin, potentially reducing the risk of root fracture [6]. Finite element analysis (FEA) has emerged as a valuable computational tool in dental biomechanics, enabling the evaluation of stress distribution and fracture resistance under simulated loading conditions [7]. FEA allows for non-destructive testing of complex biological structures and has been extensively applied in endodontics to study the biomechanical behavior of various post systems [8]. However, conventional FEA models often rely on simplified assumptions regarding material properties, boundary conditions and loading scenarios, which may limit their clinical applicability and predictive accuracy [9]. The recent integration of artificial intelligence (AI) with computational biomechanics represents a paradigm shift in dental research and clinical practice [10]. AI algorithms, particularly deep learning and machine learning approaches, can process vast amounts of data, identify complex patterns and generate highly accurate predictions that may surpass traditional analytical methods [11]. In endodontics, AI has been successfully applied for diagnosis of periapical lesions, root canal detection and treatment outcome prediction [12]. However, the combination of AI with FEA for predicting fracture patterns in post-restored ETT remains largely unexplored. Recent studies have demonstrated the potential of AI-enhanced computational models in medical and dental applications. For instance, machine learning algorithms have improved the accuracy of stress distribution predictions in orthopedic implants and dental prostheses [13]. In endodontics, AI-based image analysis has shown promise in detecting vertical root fractures and predicting treatment success rates [14]. Despite these advancements, a significant research gap exists in the application of AI-powered FEA for predicting fracture patterns in ETT restored with different post systems. Therefore, it is of interest to develop and validate an AI-powered FEA model for predicting fracture patterns in endodontically treated teeth restored with various post systems and to compare its predictive accuracy with conventional FEA methods.

## Materials and Methods:

## Study design:

This study employed a randomized controlled experimental design with a computational modeling component. The research was conducted in two phases: (1) in vitro fracture resistance testing of endodontically treated teeth restored with different post systems and (2) development and validation of an AI-powered FEA model for predicting fracture patterns.

## Sample size calculation:

Based on a pilot study, the expected difference in predictive accuracy between AI-powered and conventional FEA was 15%, with a standard deviation of 10%. Using a power analysis (α = 0.05, β = 0.20), a minimum sample size of 28 teeth per group was calculated. To account for potential attrition, 30 teeth per group were included, totaling 120 specimens.

## Tooth selection and preparation:

One hundred twenty extracted human maxillary premolars with similar dimensions (mesiodistal width: 9.0 ± 0.5 mm, buccolingual width: 8.5 ± 0.5 mm, length: 21.0 ± 1.0 mm) were selected. Teeth were stored in 0.1% thymol solution at 4°C and used within three months of extraction. Inclusion criteria included: intact crowns without caries or restorations, mature apices, absence of cracks or fractures (confirmed under 20x magnification) and similar root canal morphology. Teeth with previous endodontic treatment, root resorption, or calcified canals were excluded.

## Endodontic treatment:

All teeth underwent standardized endodontic treatment. Access cavities were prepared using diamond burs under water cooling. Working length was determined using a #10 K-file and confirmed radiographically. Canals were instrumented using ProTaper Gold rotary files (Dentsply Sirona, York, PA, USA) to size F3. Irrigation was performed with 2.5% NaOCl and 17% EDTA, followed by final rinse with distilled water. Canals were dried with paper points and obturated with gutta-percha and AH Plus sealer using the continuous wave condensation technique.

## Post space preparation and restoration:

Teeth were randomly divided into four groups (n=30) based on post type:

[1] Group 1: Fiberglass posts (RelyX Fiber Post, 3M ESPE, St. Paul, MN, USA)

[2] Group 2: Carbon fiber posts (Carbonite Post, Carbotech, Ganges, France)

[3] Group 3: Zirconia posts (CosmoPost, Ivoclar Vivadent, Schaan, Liechtenstein)

[4] Group 4: Cast metal posts (gold-palladium alloy)

Post space preparation was performed to a depth of 10 mm using Peeso reamers. Posts were cemented with dual-cure resin cement (RelyX Ultimate, 3M ESPE). Core build-up was performed using composite resin (Filtek Z250, 3M ESPE). All teeth received standardized full-coverage crowns (lithium disilicate) cemented with resin cement.

## AI-Powered FEA model development:

The AI model was developed using a deep learning approach combining convolutional neural networks (CNN) and long short-term memory (LSTM) networks. The training dataset consisted of 500 historical FEA simulations and corresponding clinical fracture outcomes from previous studies.

## The model architecture included:

[1] Input layer: Geometric and material properties of teeth and posts

[2] Feature extraction layers: CNN for spatial feature extraction

[3] Temporal analysis layers: LSTM for sequential stress analysis

[4] Output layer: Fracture probability and pattern prediction

The model was trained using TensorFlow 2.8 with Adam optimizer and categorical cross-entropy loss function. Training was performed for 500 epochs with early stopping to prevent overfitting.

## Conventional FEA modeling:

Conventional FEA models were created using ANSYS Mechanical 2022 R1. Three-dimensional models of teeth and posts were reconstructed from micro-CT scans.

## Material properties were assigned based on literature values:

[1] Dentin: E = 18.6 GPa, ν = 0.31

[2] Enamel: E = 84.1 GPa, ν = 0.33

[3] Gutta-percha: E = 0.002 GPa, ν = 0.45

[4] Fiberglass post: E = 40 GPa, ν = 0.26

[5] Carbon fiber post: E = 150 GPa, ν = 0.30

[6] Zirconia post: E = 200 GPa, ν = 0.30

[7] Cast metal post: E = 95 GPa, ν = 0.33

Mesh convergence analysis was performed to ensure element independence. A load of 300 N was applied at 45° to the long axis, simulating functional loading.

## Fracture resistance testing:

All specimens were mounted in acrylic resin blocks 2 mm below the cementoenamel junction. Fracture resistance testing was performed using a universal testing machine (Instron 5966, Norwood, MA, USA) at a crosshead speed of 1 mm/min. A 6-mm diameter steel sphere was used to apply load at 45° to the long axis on the palatal cusp. The maximum load at fracture was recorded in Newtons (N).

Fracture patterns were classified as:

[1] Favorable: Fractures above the cementoenamel junction (repairable)

[2] Unfavorable: Vertical root fractures or fractures below the cementoenamel junction (non-repairable)

## Statistical analysis:

Data were analyzed using SPSS 28.0 (IBM Corp., Armonk, NY, USA). Normality was assessed using Shapiro-Wilk test. One-way ANOVA with Tukey post-hoc test was used for multiple group comparisons. Independent t-tests compared AI-powered and conventional FEA predictions. Pearson correlation coefficients assessed relationships between predicted and actual fracture patterns. Predictive accuracy was calculated as the percentage of correct predictions. Statistical significance was set at p < 0.05.

## Results:

The AI-powered FEA model demonstrated significantly higher predictive accuracy compared to conventional FEA across all post systems (Table 1 - see PDF). Overall predictive accuracy was 92.3 ± 3.2% for AI-powered FEA versus 76.8 ± 4.5% for conventional FEA (p < 0.001). The highest accuracy for AI-powered FEA was observed with fiberglass posts (94.7 ± 2.1%), while conventional FEA showed the lowest accuracy with cast metal posts (71.2 ± 5.3%). Fracture resistance testing revealed significant differences among the post systems (Table 2 - see PDF). Fiberglass posts exhibited the highest mean fracture resistance (847.5 ± 89.3 N), followed by carbon fiber posts (812.4 ± 76.8 N). Zirconia posts showed intermediate values (756.2 ± 82.1 N), while cast metal posts demonstrated the lowest fracture resistance (698.7 ± 94.5 N). The differences between all groups were statistically significant (p < 0.05). The distribution of fracture patterns varied significantly among the post systems (Table 3 - see PDF). Fiberglass and carbon fiber posts showed predominantly favorable fracture patterns (83.3% and 76.7%, respectively), while zirconia and cast metal posts exhibited higher rates of unfavorable fractures (56.7% and 70.0%, respectively). The AI-powered FEA model accurately predicted fracture initiation sites with a correlation coefficient of r = 0.91 (p < 0.001), compared to r = 0.68 (p < 0.001) for conventional FEA. AI-powered FEA revealed distinct stress distribution patterns among the post systems. Fiberglass posts showed uniform stress distribution along the post-dentin interface, with peak stress values of 125.3 ± 12.4 MPa. Carbon fiber posts demonstrated similar patterns with slightly higher peak stresses (138.7 ± 15.2 MPa). Zirconia posts exhibited stress concentration at the coronal third of the post (187.4 ± 18.9 MPa), while cast metal posts showed the highest stress concentrations at the apical third (215.6 ± 22.3 MPa). Strong correlations were found between AI-powered FEA predictions and actual experimental outcomes. The correlation coefficient for fracture resistance prediction was r = 0.88 (p < 0.001), while for fracture pattern prediction, it was r = 0.91 (p < 0.001). Conventional FEA showed weaker correlations: r = 0.65 (p < 0.001) for fracture resistance and r = 0.68 (p < 0.001) for fracture patterns.

## Discussion:

The present study demonstrates the successful integration of artificial intelligence with finite element analysis for predicting fracture patterns in endodontically treated teeth restored with different post systems. The AI-powered FEA model significantly outperformed conventional FEA in predictive accuracy, highlighting the potential of this approach for clinical decision-making in restorative dentistry [15]. The superior performance of the AI-powered FEA model can be attributed to its ability to learn from large datasets and identify complex patterns that may not be apparent through traditional computational methods [16]. Unlike conventional FEA, which relies on predefined material properties and boundary conditions, the AI model can adapt to variations in tooth anatomy, post placement and loading conditions [17]. This adaptability is particularly valuable in dental applications, where biological variability is inherent and significant [18]. Our findings regarding fracture resistance values align with previous studies that have reported higher fracture resistance for fiber-reinforced composite posts compared to metal or ceramic posts [19]. The fiberglass posts demonstrated the highest fracture resistance (847.5 ± 89.3 N), which may be attributed to their elastic modulus being closer to that of dentin, resulting in more favorable stress distribution [20]. This observation supports the concept of biomechanical compatibility between restorative materials and tooth structure [21]. The fracture pattern analysis revealed that fiberglass and carbon fiber posts were associated with predominantly favorable (repairable) fractures, while zirconia and cast metal posts showed higher rates of unfavorable (non-repairable) fractures [22]. This finding has significant clinical implications, as unfavorable fractures often lead to tooth extraction and the need for more complex prosthetic rehabilitation [23]. The AI model's ability to accurately predict fracture patterns could help clinicians select post systems that minimize the risk of catastrophic failures [24]. The stress distribution analysis provided insights into the biomechanical behavior of different post systems. The uniform stress distribution observed with fiberglass posts suggests better load transfer along the entire post-dentin interface, reducing the risk of stress concentration at any single point [25]. In contrast, the stress concentrations observed with zirconia and cast metal posts may explain the higher incidence of unfavorable fractures in these groups [26]. These findings are consistent with previous FEA studies that have highlighted the importance of post material selection in determining stress distribution patterns [27]. The strong correlations between AI-powered FEA predictions and actual experimental outcomes (r = 0.88-0.91) demonstrate the model's reliability and potential clinical utility [28]. These correlations were significantly higher than those observed with conventional FEA, supporting the hypothesis that AI integration enhances the predictive capabilities of computational biomechanical models [29]. The ability to accurately predict both fracture resistance and fracture patterns could revolutionize treatment planning for endodontically treated teeth [30]. The clinical significance of this study extends beyond post selection. The AI-powered FEA approach could be integrated into digital workflows for comprehensive treatment planning, allowing for personalized restorative strategies based on individual patient factors [31]. Future developments may include real-time AI analysis during treatment, providing immediate feedback on optimal post placement and preparation [32]. Several limitations should be acknowledged. The in vitro nature of the study, while controlled, cannot fully replicate the complex oral environment, including factors such as occlusal forces, thermal cycling and moisture [33]. Additionally, the AI model was trained on historical data that may not encompass all possible clinical scenarios [34]. Further validation with larger datasets and clinical studies is necessary to confirm the model's generalizability [35]. Future research should focus on expanding the AI model to include additional variables such as different tooth types, restoration designs and loading conditions [36]. Longitudinal clinical studies are needed to validate the predictive accuracy of the model in real-world settings [37]. Furthermore, the integration of AI-powered FEA with other digital technologies such as cone-beam computed tomography and intraoral scanning could enhance its clinical applicability [38].

## Conclusion:

The integration of AI with finite element analysis offers a transformative approach in predicting fracture patterns of post-restored endodontically treated teeth. By enhancing accuracy and reliability, this model provides clinicians with a robust tool for optimizing post selection and treatment planning. Ultimately, such innovations may improve long-term clinical outcomes and support the preservation of natural dentition.
